# Human class I major histocompatibility complex alleles determine central nervous system injury versus repair

**DOI:** 10.1186/s12974-016-0759-4

**Published:** 2016-11-17

**Authors:** Bharath Wootla, Aleksandar Denic, Jens O. Watzlawik, Arthur E. Warrington, Laurie J. Zoecklein, Louisa M. Papke-Norton, Chella David, Moses Rodriguez

**Affiliations:** 1 Department of Neurology, Mayo Clinic, 200 First Street SW, Rochester, MN 55905 USA; 2 Mayo Clinic Center for Multiple Sclerosis and Autoimmune Neurology, Mayo Clinic, 200 First Street SW, Rochester, MN 55905 USA; 3 Center for Regenerative Medicine, Neuroregeneration, Mayo Clinic, 200 First Street SW, Rochester, MN 55905 USA; 4 Department of Neuroscience, Mayo Clinic, 4500 San Pablo Road S, Jacksonville, FL 32224 USA; 5 Department of Immunology, Mayo Clinic, 200 First Street SW, Rochester, MN 55905 USA

**Keywords:** Theiler’s murine encephalomyelitis virus, Virus persistence, Picornavirus, Human leukocyte antigen, Major histocompatibility complex

## Abstract

**Background:**

We investigated the role of human HLA class I molecules in persistent central nervous system (CNS) injury versus repair following virus infection of the CNS.

**Methods:**

Human class I A11^+^ and B27^+^ transgenic human beta-2 microglobulin positive (Hβ2m^+^) mice of the H-2^*b*^ background were generated on a combined class I-deficient (mouse beta-2 microglobulin deficient, β2m^0^) and class II-deficient (mouse Aβ^0^) phenotype. Intracranial infection with Theiler’s murine encephalomyelitis virus (TMEV) in susceptible SJL mice results in acute encephalitis with prominent injury in the hippocampus, striatum, and cortex.

**Results:**

Following infection with TMEV, a picornavirus, the Aβ^0^.β2m^0^ mice lacking active immune responses died within 18 to 21 days post-infection. These mice showed severe encephalomyelitis due to rapid replication of the viral genome. In contrast, transgenic Hβ2m mice with insertion of a single human class I MHC gene in the absence of human or mouse class II survived the acute infection. Both A11^+^ and B27^+^ mice significantly controlled virus RNA expression by 45 days and did not develop late-onset spinal cord demyelination. By 45 days post-infection (DPI), B27^+^ transgenic mice showed almost complete repair of the virus-induced brain injury, but A11^+^ mice conversely showed persistent severe hippocampal and cortical injury.

**Conclusions:**

The findings support the hypothesis that the expression of a single human class I MHC molecule, independent of persistent virus infection, influences the extent of sub frequent chronic neuronal injury or repair in the absence of a class II MHC immune response.

**Electronic supplementary material:**

The online version of this article (doi:10.1186/s12974-016-0759-4) contains supplementary material, which is available to authorized users.

## Background

The factors that control persistent injury versus repair following damage to the central nervous system (CNS) remain poorly understood. Class I major histocompatibility complex (MHC) molecules are cell surface glycoproteins that  are critical for the development of cellular immunity and are expressed in almost every nucleated cell of the body. Upon pathogen invasion, foreign peptide fragments are presented to CD8^+^ T cells via the MHC class I proteins. CD8^+^ T cells in response secrete cytokines that target and kill cells presenting specific MHC-antigen complexes. This mechanism arrests the systemic pathogen spread primarily via a perforin-mediated pathway [1]. The CNS was previously considered immune-privileged as neurons did not appear to express MHC class I molecules [2]. However, Neumann et al. demonstrated induction of class I MHC genes in cultured rat hippocampal neurons. These results demonstrated cell surface expression of MHC class I molecules in electrically silent neurons stimulated with interferon gamma [3]. More recent studies reported low level MHC class I expression in CNS neurons under physiological conditions. The importance of MHC class I molecules in neuronal development and CNS plasticity was further strengthened by the fact that neural activity regulates class I MHC gene expression in the developing and mature CNS [4]. Class I MHC molecules are also necessary for normal regressive events in the developing and adult CNS including activity-dependent synaptic weakening and structural refinement [5]. MHC class I molecules are present both pre- and post-synaptically in the visual cortex during postnatal development and in adulthood [6]; MHC class I molecules function on both sides of the synapse before, during, and after the establishment of connections in the mammalian visual cortex [7]. The expression of MHC class I is proportional to increased neural activity in the developing visual system and in the adult hippocampus after seizures [4]. Interestingly, increased expression of MHC class I is a known trigger for neurogenesis. Accordingly, both toll-like receptors expressed by adult neural stem cells and T cells also modulate neurogenesis [8–10], thus providing precedence for the hypothesis that MHC class I is involved in the development and perhaps regeneration of the CNS neurons. Finally, recent studies described the presence of functional lymphatic vessels lining the dural sinuses [11, 12]. These structures express all of the molecular hallmarks of lymphatic endothelial cells, are able to carry both fluid and immune cells from the cerebrospinal fluid, and are connected to the deep cervical lymph nodes. This suggests the brain is like every other tissue, connected to the peripheral immune system through meningeal lymphatic vessels and questions the classification of CNS as an immune-privileged organ devoid of lymphatic vasculature [2].

A large body of investigative work on CNS injury has been documented in rodent models. In contrast, studies investigating the role of the human immune responses contributing to this process are very limited. In an attempt to address this problem, we made a series of human leukocyte antigen (HLA) transgenic mice. The mice originally lacked both class I (beta-2 microglobulin deficient, β2m^0^) and class II (Aβ^0^). These mice develop normally despite the absence of endogenous CD4^+^ T cell-restricted class II immune responses or CD8^+^ T cell-restricted class I immune responses. HLA-A11 and B27 molecules were associated with viral infections [13–16] or associated with ankylosing spondylitis, reactive arthritis, uveitis, and other associated inflammatory diseases [17–19], respectively. To study the role of the human class-I immune response in CNS injury and repair, we crossed the Aβ^0^.β2m^0^ mice to express human beta-2 microglobulin (Hβ2m), which were then paired with either the A11 or B27 human class I gene to generate a functional molecule. Therefore, any immune response observed in Aβ^0^.β2m^0^.Hβ2m^+^.A11^+^ and Aβ^0^.β2m^0^.Hβ2m^+^.B27^+^ mice would be a direct consequence of the human class I gene because the class II immune response was deleted.

Infection with Theiler’s murine encephalomyelitis virus (TMEV) in different mouse strains results in two distinct phenotypes. In animals (C57BL/6 or C57BL/10 mice) that are resistant to demyelination and persistent infection, the virus during the first 10 to 12 days of infection replicates primarily in neurons of the hippocampus, striatum, cortex of the brain, and anterior horn cells of the spinal cord. In immunocompetent mice the virus is rapidly cleared from these cells irrespective of MHC haplotype in association with an intense inflammatory response [20] such that virus persistence and subsequent demyelination in the spinal cord does not occur. Oligodendrocytes and macrophages are also infected early [21]. In mice of resistant MHC haplotypes H-2^b,d,k^, no virus persistence develops and therefore no demyelination in the spinal cord ensues [22]. In animals of susceptible MHC haplotype H-2^s,v,r,u,f,q^, the virus infects neurons during the early phase similar to “resistant” mice; however, virus persists in glial cells [23] and macrophages [24–26], particularly in the spinal cord white matter and brain stem during the chronic stage (21–45 days post-infection). This results in primary demyelination, inflammation, axonal loss, and neurologic deficits that mimic human multiple sclerosis. In susceptible mice, demyelination with chronic deficits persists throughout the life of the animal.

By studying Theiler’s infection in resistant BALB/c mice, other investigators showed CD8^+^ T cells to play a protective role, possibly as suppressor cells [27, 28]. In animals susceptible to TMEV infection, activated cytotoxic T cells are generated in the brain [29] without apparent viral or myelin specificity [30]. During the chronic phase of demyelination, some susceptible strains of mice demonstrate “epitope spreading” to myelin antigens [31]. Demyelination in association with an intense inflammatory response begins in the spinal cord around day 21 following infection and is well established by day 45. Demyelination in these mice continues to worsen until approximately 90–100 days post-infection (dpi), but then reaches a plateau [32]. However, the animals continue to worsen neurologically for another subsequent 100 days after infection as a result of loss of large-diameter axons from the spinal cord, progressive spinal cord atrophy, and absence or minimal remyelination [32]. Virus persistence is detected both as RNA, antigen, and infectious particles throughout the course of the disease in a susceptible mouse. An important aspect of this model is severe injury of the hippocampus, striatum, and cortex in the early phase of infection (7 dpi), in both susceptible and resistant strain of mice, which is subsequently repaired by 45 dpi. In “resistant” strains of mice, viral RNA is cleared, whereas in “susceptible” strains of mice, viral RNA persists throughout the life of the animal. Therefore, control of persistent virus infection is not the factor that controls repair of early injury in the hippocampus, striatum, and cortex following TMEV infection.

In the current study, we investigated whether the expression of a single human class I MHC molecule will influence neuronal injury versus repair following acute viral injury of the hippocampus, striatum, and cortex. We studied the role of human class I response in the absence of a competent class II MHC immune response and also independent of persistent TMEV infection.

## Methods

### Mice

All transgenic and knockout mice were generated in the Mayo Clinic College of Medicine transgenic core facility under the direction of Dr. Chella David. All breedings were carried out in the barrier facility of the Immunogenetics mouse colony of the Mayo Clinic, Rochester, MN, USA. Aβ^0^ mice: Founder mice lacking functional mouse MHC class II molecules (Aβ^0^) were kindly provided by Drs. Diane Mathis and Christophe Benoist (INSERM, Strasbourg France; Cosgrove et al. [33]). β2m^0^ mice: Founder mice lacking functional mouse MHC class I molecules (β2m^0^) were a kind gift from Dr. Beverly Koller, University of North Carolina, Chapel Hill, NC [34]. Aβ^0^.β2m^0^ mice: The Aβ^0^ and β2m^0^ mice were mated in order to obtain Aβ^0^.β2m^0^ mice that were then bred in the Immunogenetics facility. Aβ^0^.β2m^0^.Hβ2m^+^ mice: The Aβ^0^.β2m^0^ mice were crossed to human β2m (Hβ2m^+^) transgenic mice [35] (gift of Dr. Hidde Plough, Massachusetts Institute of Technology, Boston, MA) to obtain Aβ^0^.β2m^0^.Hβ2m^+^ mice. Aβ^0^.β2m^0^.Hβ2m^+^.A11^+^ mice: transgenic HLA-A11/K^b^ (A11^+^) mice [36] were a kind gift from Dr. Jeff Alexander. The A11^+^ mice were mated to Aβ^0^.β2m^0^.Hβ2m^+^ mice in order to obtain Aβ^0^.β2m^0^.Hβ2m^+^.A11^+^ mice. All the A11 transgenic mice used in these studies were in the fifth to eighth backcross generation. Aβ^0^.β2m^0^.Hβ2m^+^.B27^+^ mice: β2m deficient mice [34] were mated with human β2m (Hβ2m^+^) transgenic mice [35]. Hβ2m^+^ littermates were intercrossed to generate a mouse having homozygous mutation of endogenous β2m gene replaced with human β2m gene (β2m^0^.Hβ2m^+^). The β2m^0^.Hβ2m^+^ mice were mated with previously described Aβ^0^.β2m^0^.B27^+^ animals [37] to obtain Aβ^0^.β2m^0^.Hβ2m^+^.B27^+^ mice. Aβ^0^.β2m^0^.B27^+^ animals lack normal expression of H2-K^*b*^ and H2-D^*b*^ and were used as negative controls during the flow cytofluorometry analysis. All the B27 transgenic mice used in these studies were in the fifth to eighth backcross generation. All animals described here bred normally and showed no abnormal signs of brain or systemic disorder. Originally, we had two founder mice for each transgenic. However, one did not breed and died, and thus, all experiments are based on one transgenic mouse for each strain. C57BL/6 (negative control that clears infection) and SJL/J (positive control that develops persistent infection and demyelination) mice were obtained from the Jackson Laboratories (Bar Harbor, Maine). Mice were followed daily until they were moribund. Mice that survived the acute infection were sacrificed at 45 dpi (endpoint of the study) for pathology and virus RNA expression.

### Screening of mice

In the absence of endogenous mouse β2m, MHC class I molecules have low expression on the cell surface. Therefore, the presence of MHC class I transgenes in β2m^0^ mice was analyzed by polymerase chain reaction (PCR). DNA was extracted from the peripheral blood according to manufacturer’s instructions using the Gentra Puregene Blood Kit (Qiagen, Germantown, MD). Four milliliters of DNA was added to 0.2 μM dNTPs, 1.0 μM each 3′ and 5′ primers in the PCR buffer in a total volume of 25 μl. Taq polymerase (0.625 U) was added to this mixture and amplified in 30 cycles under the following conditions: 3 min at 94 °C (94 °C for 1 min, annealing temp 62 °C for 1 min and 72 °C for 1 min) × 30 and 7 min at 72 °C. PCR products were analyzed by electrophoresis, and their molecular weight was compared with a standard molecular weight marker. Presence of MHC class I transgenes was identified by PCR using the following pair of oligonucleotide sequences: HLA-A11: 5′ (GGG CTC TCA CTC CAT GAG GTA TTC) and 3′ (TGT GAG TGG GCC TTC ACT TTC C); HLA-B27: 5′ (CCA CTC CAT GAG GTA TTT CCA) and 3′ (CTG TGC CTT GGC CTT GCA GA).

### Flow cytofluorometry

Human β2m, K^*b*^ and D^*b*^ identification was carried out by FACS using L-368, B8-24-3 (American Type Culture Collection, Rockville, MD) and 172-93.1 (kind gift of Dr. Günter Hammerling, DKFZ, Heidelberg) antibodies, respectively. Briefly, mononuclear cells from peripheral blood were incubated with antibodies for 30 min at 4 °C. After washing with FACS^®^ buffer (PBS containing 1% bovine serum albumin and 0.1% sodium azide) (Becton Dickinson and Co., San Jose, CA), cells were incubated with fluorescence-labeled secondary antibody (IgG goat anti-mouse Fab’2; Accurate Chemical and Scientific Corp., Westbury, NY). Expression of cell surface molecules was analyzed on 10,000 gated lymphocytes on forward and side scatter by flow cytometry.

### Virus infection and harvesting of the CNS for morphology

Transgenic mice were anesthetized and intracerebrally injected at 6 to 8 weeks of age with 2 × 10^5^ p.f.u. (plaque-forming units) of the Daniel’s strain of TMEV in a 10 μl volume. This resulted in >98% incidence of infection with rare fatalities [38]. At various times after infection, mice were perfused via intracardiac puncture with 50 ml of Trump’s fixative. Spinal cords and brains were removed and post-fixed for 24 to 48 h in Trump’s fixative in preparation for morphologic analysis.

### Spinal cord morphometry

Spinal cords were removed from spinal columns and cut into 1-mm coronal blocks. Every third block was osmicated and embedded in glycol methacrylate. Two-micron sections were prepared and stained with a modified erichrome/cresyl violet stain [39]. Morphological analysis was performed on 12 to 15 sections per mouse as previously described [40]. Briefly, each quadrant from every coronal section from each mouse was graded for the presence or absence of gray-matter disease, meningeal inflammation, and demyelination. The score was expressed as the percentage of spinal cord quadrants examined with the pathologic abnormality. A maximum score of 100 indicated a particularly pathologic abnormality in every quadrant of all spinal cord sections of a given mouse. All grading was performed without knowledge of the experimental group on coded sections. Additional spinal cord blocks were embedded in paraffin for immunocytochemistry.

### Brain pathology

Brain pathology was assessed at various time points post-infection using our previously described technique [41]. Following perfusion with Trump’s fixative, two coronal cuts were made in the intact brain at the time of removal from the skull (one section through the optic chiasm and a second section through the infundibulum). As a guide, we used the Atlas of the Mouse Brain and Spinal Cord corresponding to sections 220 and 350, page 6 [42]. This resulted in three blocks that we embedded in paraffin. This allowed for systematic analysis of the pathology of the cortex, corpus callosum, hippocampus, brainstem, striatum, and cerebellum. The resulting slides were then stained with hematoxylin and eosin. Pathologic scores were assigned without knowledge of experimental group to the following areas of the brain: cortex, corpus callosum, hippocampus, brainstem, striatum, and cerebellum. Each area of the brain was graded on a 5-point scale (0 = no pathology; 1 = no tissue destruction but only minimal inflammation; 2 = early tissue destruction [loss of architecture] and moderate inflammation; 3 = definite tissue destruction [demyelination, parenchymal damage, cell death, neurophagia, neuronal vacuolation]; 4 = necrosis [complete loss of all tissue elements with associated cellular debris]). Meningeal inflammation was assessed and graded as follows: 0 = no inflammation; 1 = one cell layer of inflammation; 2 = two cell layers of inflammation; 3 = three cell layers of inflammation; 4 = four or more cell layers of inflammation. We scored the brain region with the maximal extent of tissue damage.

### Immune-staining for human transgene proteins

Whole brains were removed from animals at 45 dpi, snap frozen, and sectioned at 10 μm in a cryostat. Acetone fixed sections were air dried and then blocked with 4% BSA:1X PBS and then incubated with anti-HLA B27 [EP-4] or anti-HLA A11 [4i93]; (Abcam Inc, Cambridge, MA). Immunoreactivity was detected using mouse on mouse HRP-Polymer (Biocare Medical, LLC, Concord, CA) and 3,3′ diaminobenzidine (Sigma #D5637).

### Immune-staining for virus protein

We performed immunocytochemistry on paraffin-embedded sections as previously described. Slides were de-paraffinized in xylene and rehydrated through an ethanol series (absolute, 95, 70, and 50%). Virus antigen staining was carried out using rabbit polyclonal antisera to TMEV-DA [43], which reacts strongly with the capsid proteins of TMEV. Following incubation with a secondary biotinylated antibody (Vector Laboratories, Burlingame, CA), immunoreactivity was detected using the avidin-biotin immunoperoxidase technique (Vector Laboratories). The reaction was developed using Hanker-Yates reagent with hydrogen peroxide as the substrate (Polysciences, Warrington, PA). Slides were lightly counterstained with Mayer’s hematoxylin. The data were expressed as the percentage of spinal cord quadrants showing virus antigen-positive cells in either the gray matter or white matter in the spinal cord.

### RNA isolation

The brain and spinal cords were removed from animals infected with TMEV. Total RNA was extracted from brain and spinal cord [44]. Briefly, the tissues were frozen and stored in liquid nitrogen. Tissues samples were homogenized in the RNA STAT-60 (1 ml/100 mg tissue) (TEL-TEST, INC., Friendswood, TX) with a homogenizer, and total RNA was isolated according to the manufacturer’s recommendations. The RNA concentrations were determined by spectrophotometer. The RNA samples were equilibrated to a concentration of 0.25 μg/μl and stored at −80 °C.

### RT-PCR and real-time analysis for virus RNA

The VP2 fragment of TMEV, a viral capsid region of DA virus, was amplified by RT-PCR using gene-specific primers [44]. The primer pair sequences for VP2 of DA virus were as follows: forward (5′-TGGTCGACTCTGTGGTTACG-3′) and reverse (5′-GCCGGTCTTGCAAAGATAGT-3′). Gluceraldehyde-3-phosphate dehydrogenase (GAPDH) was used as a control for inter-sample variability. The sequences used for assaying the presence of GAPDH were as follows: forward (5′-ACCACCATGGAGAAGGC-3′) and reverse (5′-GGCATGGACTGTGGTCATGA-3′). Sizes of PCR products amplified with primers were 238 base pairs for VP2 and 236 base pairs for GAPDH.

Gene-copy standards were generated with each set of samples. Standards were generated by serial tenfold dilutions of plasmid cDNA. Standards were amplified in parallel with unknown samples by real-time quantitative RT-PCR using the LightCycler (Roche, Indianapolis, IN). We used LightCycler 3 software analysis to generate standard curves. Negative controls (omitting input cDNA) were also used in each PCR run to confirm the specificity of the PCR products. PCR product curves were linear across serial tenfold dilutions, and the melting curve analysis indicated synthesis of a single homogenous product of the expected melting temperature. The reactions were done in 20-μl capillaries 7.0 mM Mg^2+^, 10 pM concentrations of each forward and reverse primer, 4.0 μl of LightCycler-RT-PCR Reaction Mix SYBR Green I (LightCycler-RNA Amplification Kit SYBR Green I; Roche), 2 μl of resolution solution, 0.4 μl of LightCycler-RT-PCR Enzyme Mix, sterile H_2_O, and 0.5 μg total RNA. Reaction conditions for RT-PCR for VP2 and GAPDH were as follows: reverse transcription at 55 °C for 10 min, followed by denaturation at 95 °C for 2 min, followed by 40 cycles of amplification. Amplification conditions were as follows: denaturing at 95 °C at 20 °C/s without plateau phase, annealing at 57 °C for 7 s, and extension 72 °C for 15 s. The accumulation of products was monitored by SYBR Green fluorescence at the completion of each cycle. There was a direct relationship between the cycle number at which accumulation of PCR products became exponential and the log concentration of RNA molecules initially present in the RT-PCR reaction. The reaction conditions for melting curve analysis were as follows: denaturation to 95 °C at 20 °C/s without plateau phase, annealing at 60 °C for 5 s, and denaturation to 95 °C at 0.1 °C/s, with continuous monitoring of SYBR Green fluorescence. RNA samples (*n* = 76) from DA virus-infected mice were analyzed for GAPDH mRNA levels to determine the levels of mRNA per sample and the technical reproducibility. The GAPDH mRNA level per sample was log_10_ 7.19 ± 0.02 (mean ± the SEM). Therefore, the marked variations in viral RNA levels in individual specimens could not be attributed to differences in amplifiable material. The amount of viral RNA was expressed as log_10_ virus copy number/0.5 μg RNA total. We used viral RNA as the primary assay for virus infection since plaque assays are difficult and many times unreliable with Theiler’s virus [45]. Previous work from our lab has shown an excellent correlation between virus RNA and plaque assay [46].

### Virus-specific antibody isotype ELISA

Whole blood was collected from mice at time of sacrifice, and sera was isolated and stored at –80 °C until further use. Total serum IgG against TMEV was assessed by enzyme-linked immunosorbent assay (ELISA) as described [47]. Purified virus was adsorbed on 96-well plates (Immulon II; Dynatech Laboratories Inc., Chantilly, VA) and then blocked with 1.0% bovine serum albumin (BSA; Sigma Chemical Co. St. Louis, MO) in PBS. Serial dilutions of sera were made in 0.2% BSA/PBS and added in triplicate. Biotinylated anti-mouse IgG secondary antibodies were used for detection (Jackson Immunoresearch Labs, Westbury, NY). Signals were amplified with streptavidin-labeled alkaline-phosphatase (Jackson Immunoresearch Labs) and detected using p-nitrophenyl phosphate as the substrate. Absorbances were read at 405 nm and plotted as a function of serum dilution factors.

### Statistics

Data were analyzed using either the Student’s *t* test for normally distributed data or the Mann-Whitney rank sum test for data that were not normally distributed. ANOVA was used for comparisons of more than one group. The Siegel-Tukey test was used for all pair-wise multiple comparison procedures. Proportional data were evaluated using the *z*-test. The level for significance was set as *P* < 0.05 for all tests.

## Results

### Derivation of transgenic mice and expression of MHC class I molecules

**Fig. 1:**
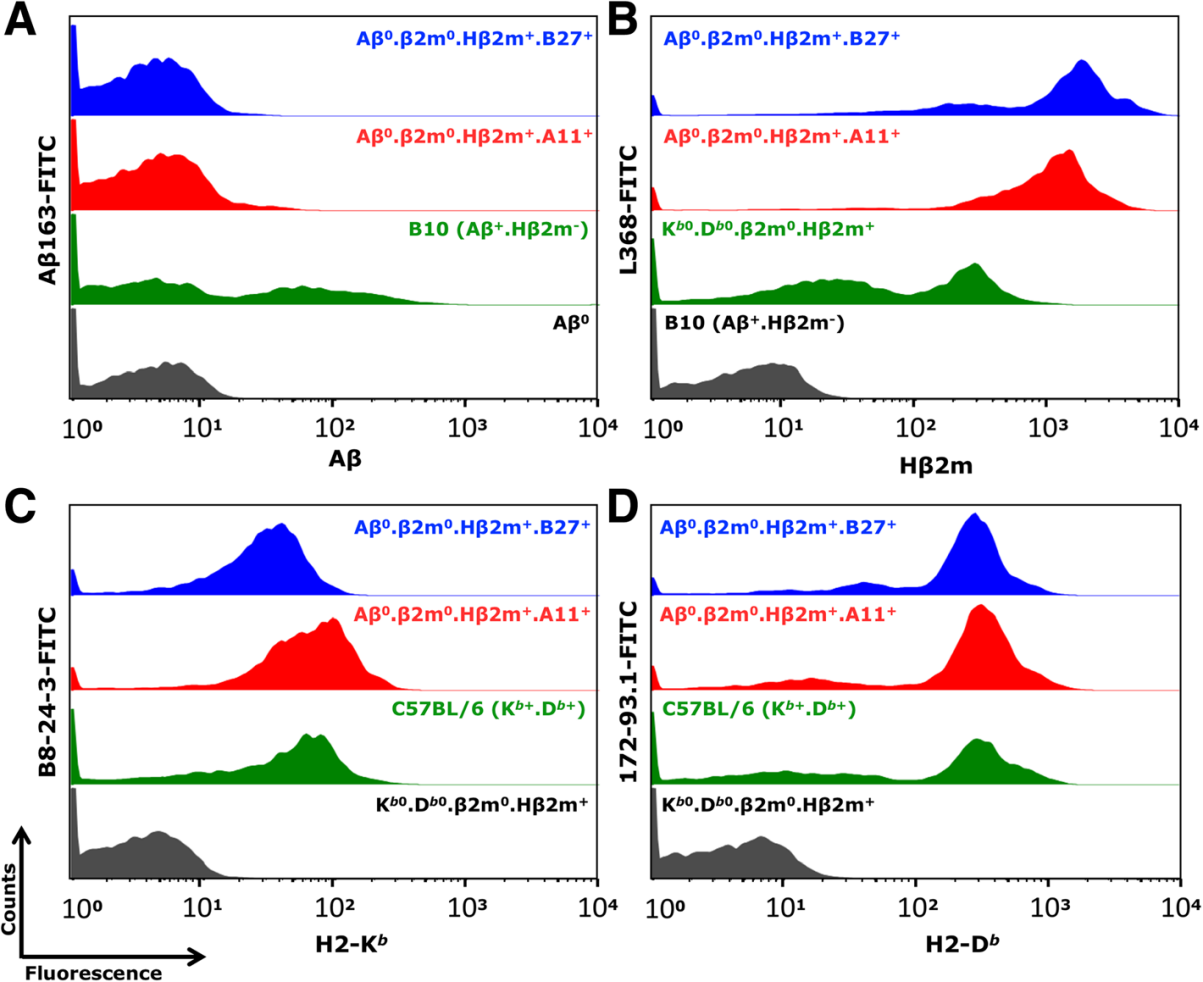
Flow cytometric analysis in Aβ^0^.β2m^0^.Hβ2m^+^.A11^+^ and Aβ^0^.β2m^0^.Hβ2m^+^.B27^+^ mice. Cell surface expression was examined by antibodies to Aβ (Aβ-163), Hβ2m (L-368), H2-K^*b*^ (B8-24-3) and H2-D^*b*^ (172-93.1). Controls were B10 (Aβ^+^.Hβ2m^−^), Aβ^0^, K^*b*0^.D^*b*0^.β2m^0^.Hβ2m^+^, and C57BL/6 (K^*b*+^.D^*b*+^) mice. In each panel, the *gray* histogram represents negative control, the *green* histogram represents a positive control, and the *red* and *blue* histograms represent Aβ^0^.β2m^0^.Hβ2m^+^.A11^+^ and Aβ^0^.β2m^0^.Hβ2m^+^.B27^+^ mice, respectively. Non-expression of Aβ (**a**) and expression of Hβ2m (**b**), H2-K^*b*^ (**c**), and H2-D^*b*^ (**d**) in transgenic human class I mice is shown

Aβ^0^, β2m^0^, and Aβ^0^.β2m^0^ mice were obtained as described in the “Methods” section. Aβ^0^.β2m^0^ mice have no functional class II (CD4^+^ T cells) or class I (CD8^+^ T cells) immune responses. To simulate expression of human HLA molecules on cell surface, we replaced mouse endogenous β2m with human β2m transgene and generated Aβ^0^.β2m^0^.Hβ2m^+^. These mice were then subsequently crossed with mice expressing the HLA-A11 transgene to obtain Aβ^0^.β2m^0^.Hβ2m^+^.A11^+^ mice. β2m^0^.Hβ2m^+^ mice were mated with HLA-B27 transgene positive mice (β2m^0^.B27^+^) to generate β2m^0^.Hβ2m^+^.B27^+^ mice. These were then crossed with Aβ^0^ mice to obtain Aβ^0^.β2m^0^.Hβ2m^+^.B27^+^ mice. The Aβ^0^.β2m^0^.Hβ2m^+^.A11^+^ and Aβ^0^.β2m^0^.Hβ2m^+^.B27^+^ mice did not show expression of Aβ^0^ (Fig. 1a). We also noted that expression of human β2m (Fig. 1b) in the context of transgenes HLA-A11 and HLA-B27 restored normal expression of endogenous mouse class I (H2-K^*b*^ and H2-D^*b*^) molecules. The level of expression of H2-K^*b*^ and H2-D^*b*^ was similar between Aβ^0^.β2m^0^.Hβ2m^+^.A11^+^ and Aβ^0^.β2m^0^.Hβ2m^+^.B27^+^ mice (Fig. 1c, d). The presence of MHC class I transgenes A11 and B27 was identified by PCR using oligonucleotide sequences described in the “Methods” section. Immunostaining of brain sections confirmed the expression of A11 (Additional file 1: Figure S1A, B) or B27 (Additional file 1: Figure S1C, D). However, the level of immunostaining in brain sections was very low as seen by the figures because of the difficulty of finding antibodies that worked well for immunocytochemistry and the low level of MHC normally observed in CNS.

### Transgenic expression of human class I A11^+^ or B27^+^ gene prevents death of TMEV-infected Aβ^0^.β2m^0^.Hβ2m^+^ mice

Previous experiments performed with Aβ^0^.β2m^0^ mice demonstrated that these mice die of overwhelming virus-induced encephalomyelitis within 16 to 18 dpi with TMEV [48]. We infected Aβ^0^.β2m^0^ mice (*N* = 25) with TMEV, and mice were either moribund or dead by 18 dpi. As controls, we infected β2m^0^ mice (*N* = 27), Aβ^0^ mice (*N* = 38), and C57BL/6 mice (*N* = 26) that showed only mild early neurologic deficits, and all survived past 18 dpi.

We then asked whether the expression of a single class I human HLA transgene in lieu of mouse class I would protect mice from death. We hypothesized that substitution of the mouse class I gene with the human class I gene would generate a line of mice that are similar to Aβ^0^ mice. We infected Aβ^0^.β2m^0^.Hβ2m^+^.A11^+^ (*N* = 72), Aβ^0^.β2m^0^.Hβ2m^+^.B27^+^ (*N* = 29), and Aβ^0^.β2m^0^.Hβ2m^+^ (*N* = 11) mice with TMEV. As expected, all mice survived past the 18-day time point with minimal early neurologic deficits. We chose to sacrifice these human class I transgenic MHC mice at 7 dpi (time of maximal viral replication and early CNS pathology) or 45 dpi for pathologic and virology analyses. We decided to examine surviving mice at 45 dpi, because this time point has been used previously in past studies [49] to determine viral persistence and spinal cord demyelination, when mice are entering the chronic phase of infection.

### Expression of human class I MHC transgenes prevents severe early neuronal gray matter disease observed in TMEV-infected Aβ^0^.β2m^0^ mice

Having observed that expression of human class I MHC transgene protects Aβ^0^.β2m^0^ mice from death following a lethal dose of virus in the mouse CNS, we subsequently examined spinal cord pathology (Table 1). Spinal cord sections were studied at 18 to 21 dpi. We quantified the percentage of spinal cord quadrants with neuronal injury in the gray matter and demyelination and inflammation in the white matter. Gray matter disease was characterized by neuronal loss, intense inflammation around anterior horn cells, and vacuolar degeneration of neurons. The number of spinal cord quadrants with neuronal injury particularly in the gray matter were increased in Aβ^0^.β2m^0^ mice (13.0 ± 3.1, *N* = 21) compared to the gray matter of immunocompetent C57BL/6 mice (0.0 ± 0.0, *N* = 11). The decrease in the gray matter disease in C57BL/6 mice compared to Aβ^0^.β2m^0^ mice was statistically significant (*P* = 0.005, rank sum test). Similar (low) levels of gray matter spinal cord pathology was observed in β2m^0^ mice (1.3 ± 0.6, *N* = 11) and in Aβ^0^ mice (0.0 ± 0.0, *N* = 13). Meningeal inflammation was observed in Aβ^0^.β2m^0^ (4.4 ± 1.8, *N* = 21) and β2m^0^ (3.4 ± 0.7, *N* = 11) mice, whereas no inflammation was observed in C57BL/6 (0.0 ± 0.0, *N* = 11) mice or Aβ^0^ mice (0.0 ± 0.0, *N* = 13). The difference in meningeal inflammation was statistically significant (*P* < 0.001, ANOVA on ranks), when comparing Aβ^0^.β2m^0^, Aβ^0^, β2m^0^, and C57BL/6 mice.

**Table 1:** Spinal cord pathology of TMEV-infected mice (45 dpi)

Strain	Number	Gray matter disease	Meningeal inflammation
Aβ^0^	19	0.5 ± 0.3	1.3 ± 0.7
β2m^0^	7	0.5 ± 0.3	9.7 ± 4.0
Aβ^0^.β2m^0^. Hβ2m^+^.A11+	16	0.1 ± 0.1	0.9 ± 0.4
Aβ^0^.β2m^0^. Hβ2m^+^.B27+	11	0.0 ± 0.0	0.4 ± 0.4
C57BL/6	18	0.0 ± 0.0	0.0 ± 0.0
SJL/J	17	0.4 ± 0.3	23.6 ± 3.7

Data expressed as the percent of spinal cord quadrants showing the pathologic abnormality (mean ± SEM)

Next, we studied mice with human class I transgenes infected with TMEV for 21 days. The extent of gray-matter pathology in the spinal cord at 21 days was 0.1 ± 0.1 for Aβ^0^.β2m^0^.Hβ2m^+^.A11^+^ (*N* = 7) mice and 0.0 ± 0.0 for Aβ^0^.β2m^0^.Hβ2m^+^.B27^+^ (*N* = 5). Therefore, transgenic insertion of a human class I gene almost completely eliminated neuronal injury in the spinal cord during the early phase of disease. Similarly, the extent of inflammation (Aβ^0^.β2m^0^.Hβ2m^+^.A11^+^ [0.3 ± 0.3] or Aβ^0^.β2m^0^.Hβ2m^+^.B27^+^ [0.0 ± 0.0]) or demyelination (Aβ^0^.β2m^0^.Hβ2m^+^.A11^+^ [0.0 ± 0.0] and Aβ^0^.β2m^0^.Hβ2m^+^.B27^+^ [0.0 ± 0.0]) in the class I transgenic mice was low or completely absent. Therefore, the survival of mice as a result of the insertion of the human class I transgenes was associated with minimal pathology in the spinal cord gray matter and minimal demyelination in the white matter during the early disease phase.

### Expression of A11^+^ or B27^+^ human class I transgene protects mice from spinal cord demyelination

**Fig. 2:**
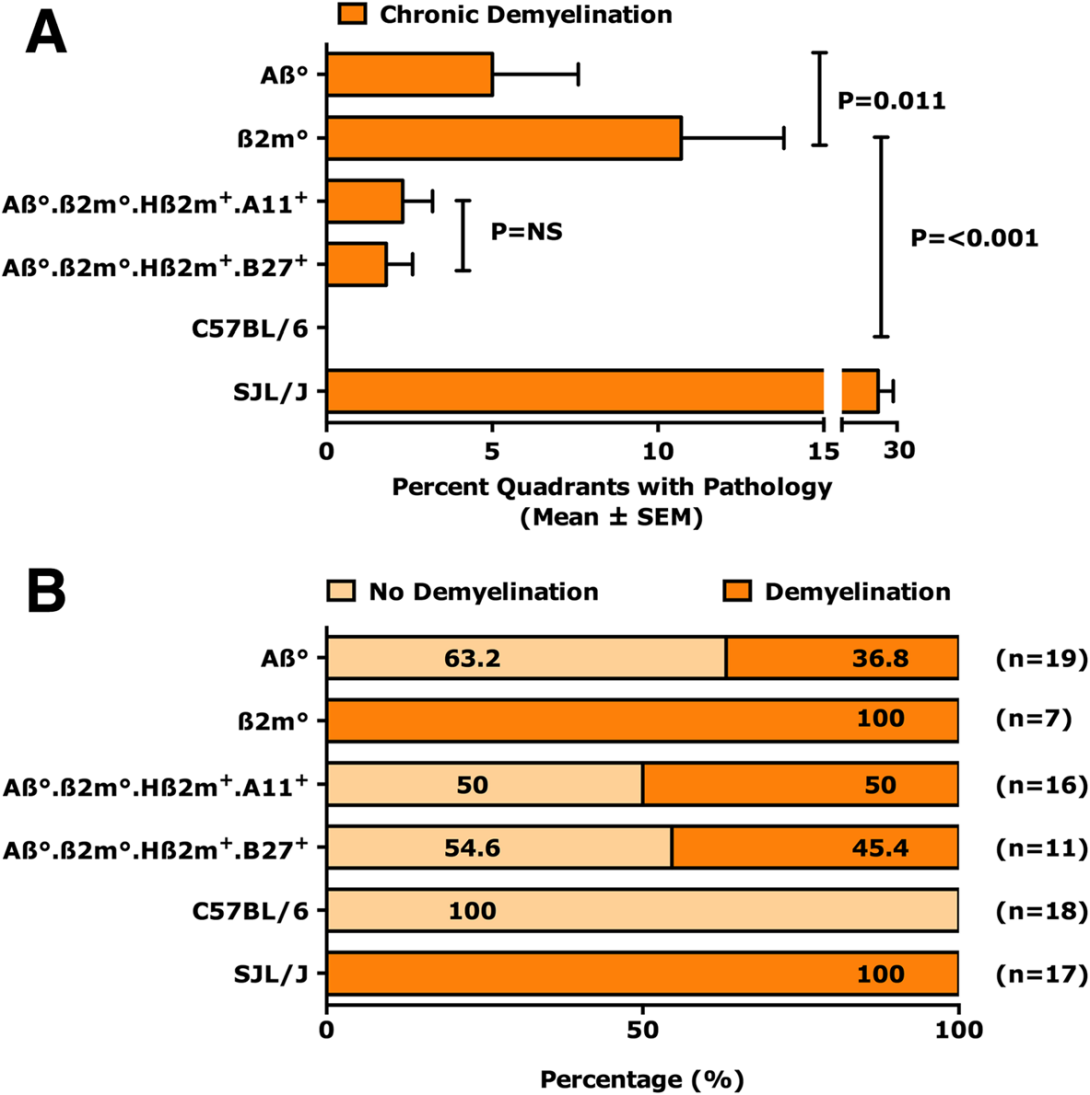
**a** Chronic demyelination scores in spinal cords of different transgenic mice compared to wild-type mice. Morphological analysis of spinal cords from mice sacrificed at 45 dpi was performed on 12 to 15 sections per mouse. Each quadrant from every coronal section of each mouse was graded for demyelination. Percentage of spinal cord quadrants examined with the pathologic abnormality scores are shown. The number of mice analyzed in the experiment is listed beside each bar. **b** Percentage of animals positive for demyelination. Number of animals positive for demyelination based on morphological analysis are plotted as percentage positive for demyelination in each group. One hundred percent of mice in the susceptible phenotype showed demyelination in the sections tested, whereas mice with competent immune system did not show any demyelination

Having established that expression of either A11^+^ or B27^+^ transgenes protects Aβ^0^.β2m^0^ mice from acute spinal cord gray-matter disease, we asked whether the expression of these human genes would influence demyelination at 45 dpi (Fig. 2a). Aβ^0^.β2m^0^ mice were not available for these analyses, since none survived to this time point. As described previously, both β2m^0^ mice [40] and Aβ^0^ mice [47] showed demyelination in the spinal cord white matter. The percent (±SEM) of spinal cord quadrants with demyelination was 10.7 ± 3.1 for β2m^0^ mice (*N* = 7) and 5.0 ± 2.6 for Aβ^0^ mice (*N* = 19). Immunocompetent C57BL/6 mice (*N* = 18) of resistant haplotype showed no demyelination (0.0 ± 0.0). The difference in demyelination between β2m^0^ mice and C57BL/6 mice was statistically significant (*P* < 0.001, Mann-Whitney rank sum test). Similarly, the increase in demyelination in β2m^0^ mice compared to Aβ^0^ mice was statistically significant (*P* = 0.011, Mann-Whitney rank sum test). As a positive control, we used highly susceptible SJL/J mice infected for 45 days that showed significant meningeal inflammation (23.6 ± 3.7, *N* = 17) and demyelination (24.9 ± 4.1, *N* = 17) in the spinal cord. At 45 dpi (Fig. 2a), only a few spinal cord quadrants showed demyelination in Aβ^0^.β2m^0^.Hβ2m^+^.A11^+^ (2.3 ± 0.9, *N* = 16) or Aβ^0^.β2m^0^.Hβ2m^+^.B27^+^ (1.8 ± 0.8, *N* = 11) mice, as compared to susceptible SJL/J mice (24.9 ± 4.1, *N* = 17). There was no statistical difference in the number of spinal cord quadrants with demyelination when comparing Aβ^0^.β2m^0^.Hβ2m^+^.A11^+^, Aβ^0^.β2m^0^.Hβ2m^+^.B27^+^ or Aβ^0^ mice (ANOVA or ranks, Fig. 2b). In addition, there was no significant difference in the number of spinal quadrants with demyelination in Aβ^0^.β2m^0^.Hβ2m^+^.A11^+^ mice as compared to Aβ^0^.β2m^0^.Hβ2m^+^.B27^+^ mice at 45 days after infection (*P* = 0.767, rank sum test, Fig. 2b). We considered mice with less than 5% of the quadrants positive for demyelination as resistant for spinal cord demyelination and virus persistence, in contrast to susceptible strains which usually show greater than 20% of the quadrants positive for demyelination.

### Expression of A11 results in the persistence of brain injury, whereas expression of B27 results in brain repair

**Fig. 3:**
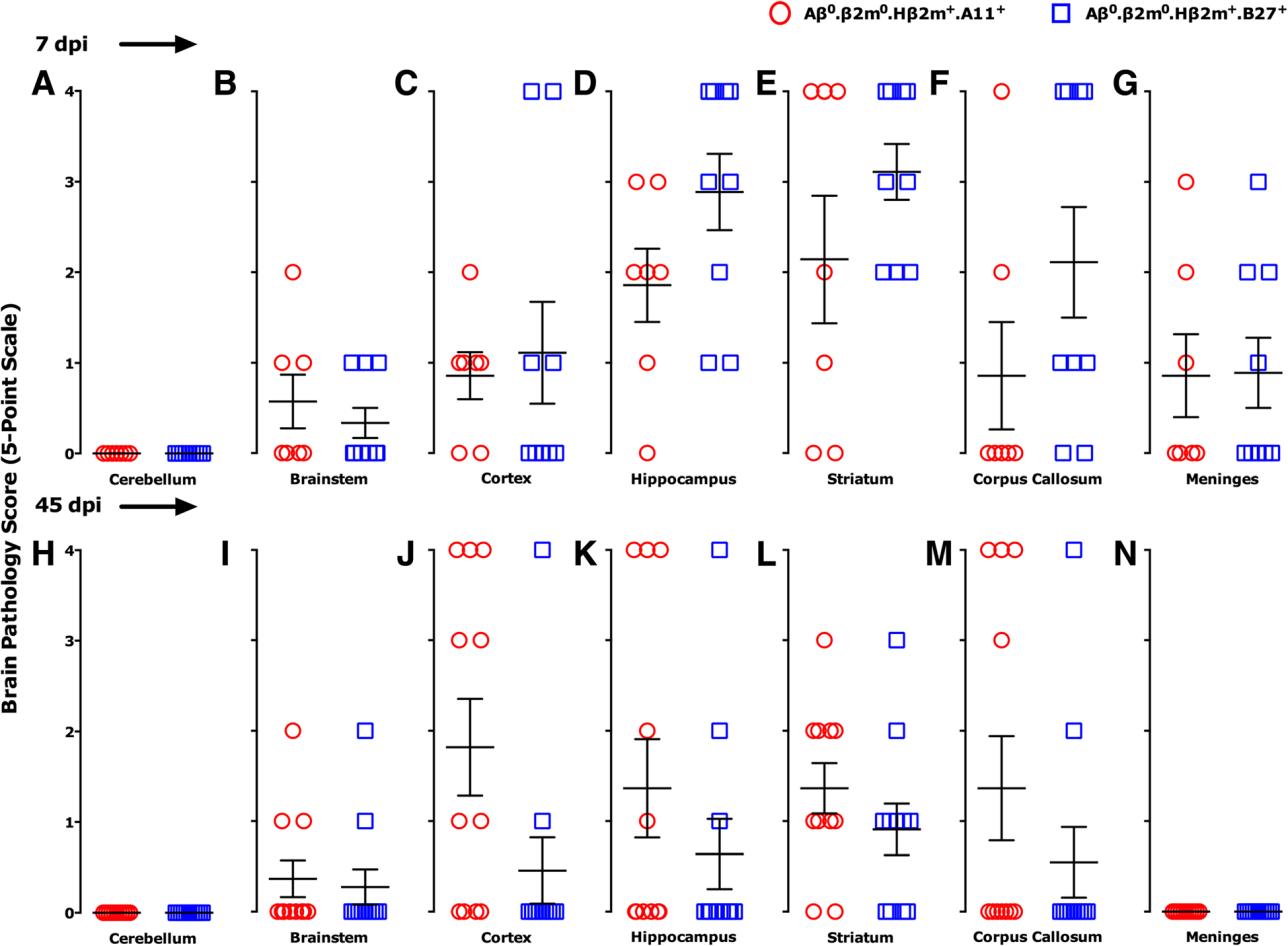
Pathology scores of the brain. Pathologic analysis of brain areas (cerebellum, brain stem, cortex, hippocampus, striatum, corpus callosum, and meninges) at 7 days (**a**–**g**) and 45 days (**h**–**n**) following TMEV infection (dpi). Mouse strains shown are Aβ^0^.β2m^0^.Hβ2m^+^.A11^+^ (*red open circle*) and Aβ^0^.β2m^0^.Hβ2m^+^.B27^+^ (*blue open square*). Pathologic qualitative scores from 0 to 4 are described in the materials and methods. Each symbol represents one mouse. There were no differences between the two strains in the distribution of brain pathology at 7 days after infection (**a**–**g**). At the 45-day time point, there was less brain pathology in Aβ^0^.β2m^0^.Hβ2^+^.B27^+^ mice as compared to Aβ^0^.β2m^0^.Hβ2m^+^.A11^+^mice (*P* = 0.027, rank sum test; **h**–**n**)

We investigated whether expression of A11 or B27 protected specific populations of brain cells from injury. Using a 5-point scale (materials and methods), we first assessed brain pathology at 7 dpi (Fig. 3a–g) to compare the degree and extent of brain injury between the strains. Scatter-point analyses of the brain pathology from Aβ^0^.β2m^0^.Hβ2m^+^.A11^+^ and Aβ^0^.β2m^0^.Hβ2^+^.B27^+^ strains showed similar pathology in the cortex, hippocampus, striatum, and corpus callosum with minimal disease in the cerebellum. Meningeal inflammation was similar in both strains. Aβ^0^.β2m^0^.Hβ2m^+^.B27^+^ strain had a higher predisposition to brain pathology in the corpus callosum, striatum, and brain stem. For quantitative and statistical comparisons, we compiled all pathology brain scores into data sets segregated by strain and days after infection. There was no statistical difference (*P* = 0.194, rank sum test) in the pathologic scores at 7 days for Aβ^0^.β2m^0^.Hβ2m^+^.A11^+^ mice (1.020 ± 0.186, *N* = 49) compared to Aβ^0^.β2m^0^.Hβ2m^+^.B27^+^ mice (1.492 ± 0.205, *N* = 63). Following the survival of Aβ^0^.β2m^0^.Hβ2m^+^.A11^+^ and Aβ^0^.β2m^0^.Hβ2m^+^.B27^+^ mice past the 18-to-21 dpi as compared to immune-deficient Aβ^0^.β2m^0^ mice, we asked whether this phenomenon reflected the extent of brain pathology. At 18 to 21 dpi, the brain pathologic scores in Aβ^0^.β2m^0^ mice (1.714 ± 0.181, *N* = 56) were significantly greater than in Aβ^0^.β2m^0^.Hβ2m^+^.A11^+^ mice (0.796 ± 0.193, *N* = 49, *P* < 0.001, rank sum test) or for Aβ^0^.β2m^0^.Hβ2m^+^.B27^+^ (0.943 ± 0.232, *N* = 35, *P* = 0.004, rank sum test). Therefore, we concluded that the expression of a human class I gene protected Aβ^0^.β2m^0^ mice from lethal encephalitis.

**Fig. 4:**
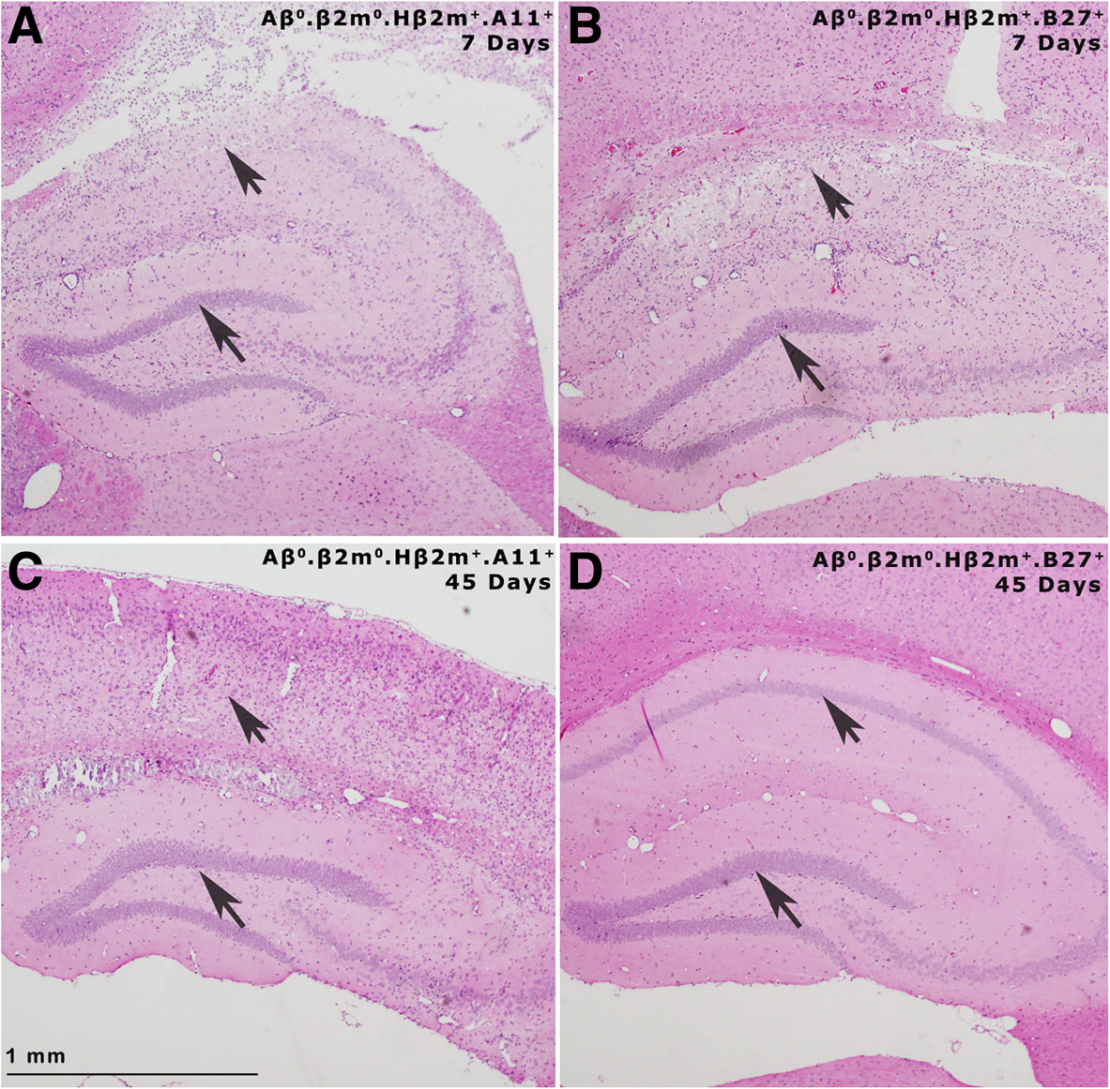
Pathology of the hippocampus. Paraffin sections stained with hematoxylin and eosin showing the hippocampus following virus infection. *Arrowheads* point to the dentate gyrus of the hippocampus that is preserved in all strains. *Arrows* point to the pyramidal cells of the hippocampus, which is destroyed in both B27+ and A11+ strains at day 7 DPI but is repaired in B27+ mice by 45 dpi but not repaired in A11+ strains. **a** Aβ^0^.β2m0.Hβ2m^+^.A11^+^ mouse at 7 dpi. **b** Aβ^0^.β2m^0^.Hβ2m^+^.B27^+^ mouse at 7 dpi. **c** Aβ^0^.β2m^0^.Hβ2m^+^.A11^+^ mouse at 45 dpi. **d** Aβ^0^.β2m^0^.Hβ2m^+^.B27^+^ mouse at 45 dpi. Note extensive shrinkage of the brain parenchyma in the Aβ^0^.β2m^0^.Hβ2m^+^.A11^+^ mouse at 45 dpi compared to the Aβ^0^.β2m^0^.Hβ2m^+^.B27^+^ mouse. *Scale bar* = 1 mm

We then addressed whether a particular human class I MHC allele would influence persistent brain injury versus repair in the CNS following the early injury in both strains in the hippocampus, striatum, and cortex. We analyzed the distribution of brain disease at 45 dpi. The difference in the pathological scores of the hippocampus and the cortex was substantial between these strains. There was similar severe loss of pyramidal cell neurons in the hippocampus of both strains at 7 days after infection (Fig. 4a, b). Many areas showed frank necrosis with complete loss of neuronal architecture (brain pathology scores of 4, Fig. 3j, k, m). At 45 dpi in Aβ^0^.β2m^0^.Hβ2m^+^.B27^+^ mice, there was essentially complete repair of the pyramidal cell layer of the hippocampus (Fig. 4d). In contrast, the loss of pyramidal cells persisted in Aβ^0^.β2m^0^.Hβ2m^+^.A11^+^ mice (Fig. 4c). Similar findings were observed in the cerebral cortex, where Aβ^0^.β2m^0^.Hβ2m^+^.B27^+^ mice showed normal cortical neurons at 45 dpi, whereas Aβ^0^.β2m^0^.Hβ2m^+^.A11^+^ mice showed cortical shrinkage as a manifestation of neuronal loss. There was more brain pathology (Fig. 3h–n) at 45 dpi (*P* = 0.027, rank sum test) in Aβ^0^.β2m^0^.Hβ2m^+^.A11^+^ mice (0.964 ± 0.137, *N* = 112) than in Aβ^0^.β2m^0^.Hβ2m^+^.B27^+^ mice (0.403 ± 0.198, *N* = 77). These findings support the hypothesis that the presence of the B27 allele results in a class I-restricted immune response favoring repair. In contrast, the presence of the A11 allele results in a class I-restricted immune response favoring persistent injury. To show the quantitative effect on the human class I MHC genes in injury and repair present in the hippocampus and striatum, we compared the combined pathologic scores in these areas between Aβ^0^.β2m^0^.Hβ2m^+^.A11^+^ and Aβ^0^.β2m^0^.Hβ2m^+^.B27^+^ mice. We found a borderline significance (*P* = 0.05, rank sum test) for the increased extent of pathology in the hippocampus and striatum of Aβ^0^.β2m^0^.Hβ2m^+^.A11^+^ mice (1.656 ± 0.256, *N* = 32) as compared to Aβ^0^.β2m^0^.Hβ2m^+^.B27^+^ mice (0.917 ± 0.255, *N* = 24).

### Aβ^0^.β2m^0^.Hβ2m^+^.A11^+^ and Aβ^0^.β2m^0^.Hβ2m^+^.B27^+^ transgenic mice show a similar distribution of virus protein in the brain during the early and late disease stages

We asked whether failure to control virus antigen expression in the brain resulted in the slow and diminished repair of hippocampus, striatum, and cortex of the Aβ^0^.β2m^0^.Hβ2m^+^.A11^+^ mice compared to Aβ^0^.β2m^0^.Hβ2m^+^.B27^+^ mice (Fig. 3). Animals of the resistant H-2^*b*^ haplotype replicate TMEV in brain from 7 to 21 days after intracerebral inoculation. Virus antigen is expressed abundantly in the hippocampus and cortex but to a lesser extent in striatum and brain stem. Expression levels of viral antigens decrease significantly in the brain by 45 dpi such that only a few antigen-positive cells are observed. During the early infection of the brain, neurons are the primary targets of infection. However, a few oligodendrocytes, astrocytes, and macrophages also express virus antigen.

**Fig. 5:**
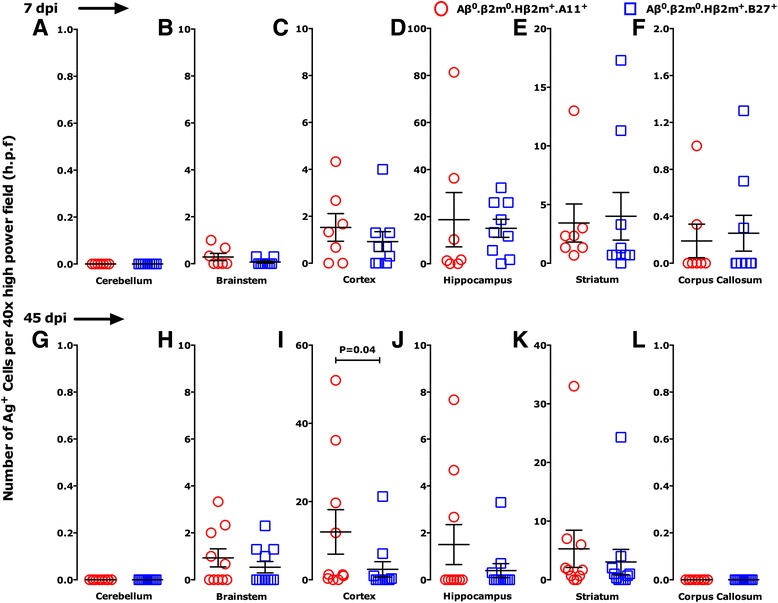
Virus antigen-positive cells in different brain areas. Brain areas (cerebellum, brain stem, cortex, hippocampus, striatum, and corpus callosum) at 7 days (**a**–**f**) and 45 days (**g**–**l**) following TMEV infection (dpi) were quantified for a number of virus antigen-positive cells per ×40 high-power field (h.p.f) in area of maximal pathology. There were similar numbers of virus antigen-positive cells across the brain regions at both 7 and 45 dpi (except the cortex at 45 dpi, **i**)

We quantified the number of virus antigen-positive cells per ×40 high-power field (h.p.f.) in the cerebellum, brain stem, cortex, hippocampus, striatum, and corpus callosum of Aβ^0^.β2m^0^.Hβ2m^+^.A11^+^ and Aβ^0^.β2m^0^.Hβ2m^+^.B27^+^ mice at 7 (Fig. 5a–f) and 45 dpi (Fig. 5g–l). We chose these time points because of the similar brain pathology in both strains at 7 dpi, whereas at 45 dpi brain pathology was significantly higher in Aβ^0^.β2m^0^.Hβ2m^+^.A11^+^ mice compared to the Aβ^0^.β2m^0^.Hβ2m^+^.B27^+^ mice. We quantitated virus antigen-positive cells in areas of maximal pathology (5 to 11 specimens per experimental group). There were no significant differences in the number of virus antigen-positive cells across the brain regions at 7 dpi. Similar numbers of virus antigen-positive cells were observed in the all the other regions of the brain of both strains. Only a few antigen-positive cells were observed in the cerebellum and brain stem, since these areas demonstrated minimal pathology.

It was critical to discern whether the minimal repair observed in Aβ^0^.β2m^0^.Hβ2m^+^.A11^+^ mice was the result of virus antigen persistence in brain areas with injury. Analysis of five Aβ^0^.β2m^0^.Hβ2m^+^.A11^+^ mice with severe CNS pathology at 45 dpi showed minimal antigen-positive cells in the brain areas examined (Fig. 5g–l). The number of virus antigen-positive cells were not significantly different in the majority of areas examined comparing Aβ^0^.β2m^0^.Hβ2m^+^.A11^+^ mice to Aβ^0^.β2m^0^.Hβ2m^+^.B27^+^ mice at 45 dpi. This was the case even though four of five mice had pathological scores of “4” in striatum and/or hippocampus (Fig. 3j, k, l). However, more virus antigen-positive cells were quantified in the cortex of Aβ^0^.β2m^0^.Hβ2m^+^.A11^+^ mice (12.2 ± 5.7) compared to the Aβ^0^.β2m^0^.Hβ2m^+^.B27^+^ mice (2.7 ± 2.0) and this was statistically significant (*P* = 0.04, rank sum test). This may not be biologically significant as quantification from just one mouse influenced this result (Fig. 5i). In addition, Aβ^0^.β2m^0^.Hβ2m^+^.B27^+^ mice with resolved pathology in the striatum and hippocampus also showed minimal virus antigen expression in these areas. We concluded that persistent virus antigen expression in CNS cells likely did not delay recovery of CNS pathology in Aβ^0^.β2m^0^.Hβ2m^+^.A11^+^ mice. Therefore, it is possible factors other than virus clearance are important in class I MHC-mediated CNS repair.

### Aβ^0^.β2m^0^.Hβ2m^+^.A11^+^ and Aβ^0^.β2m^0^.Hβ2m^+^.B27^+^ transgenic mice propagate similar extent of viral RNA in the brain during TMEV infection

**Fig. 6:**
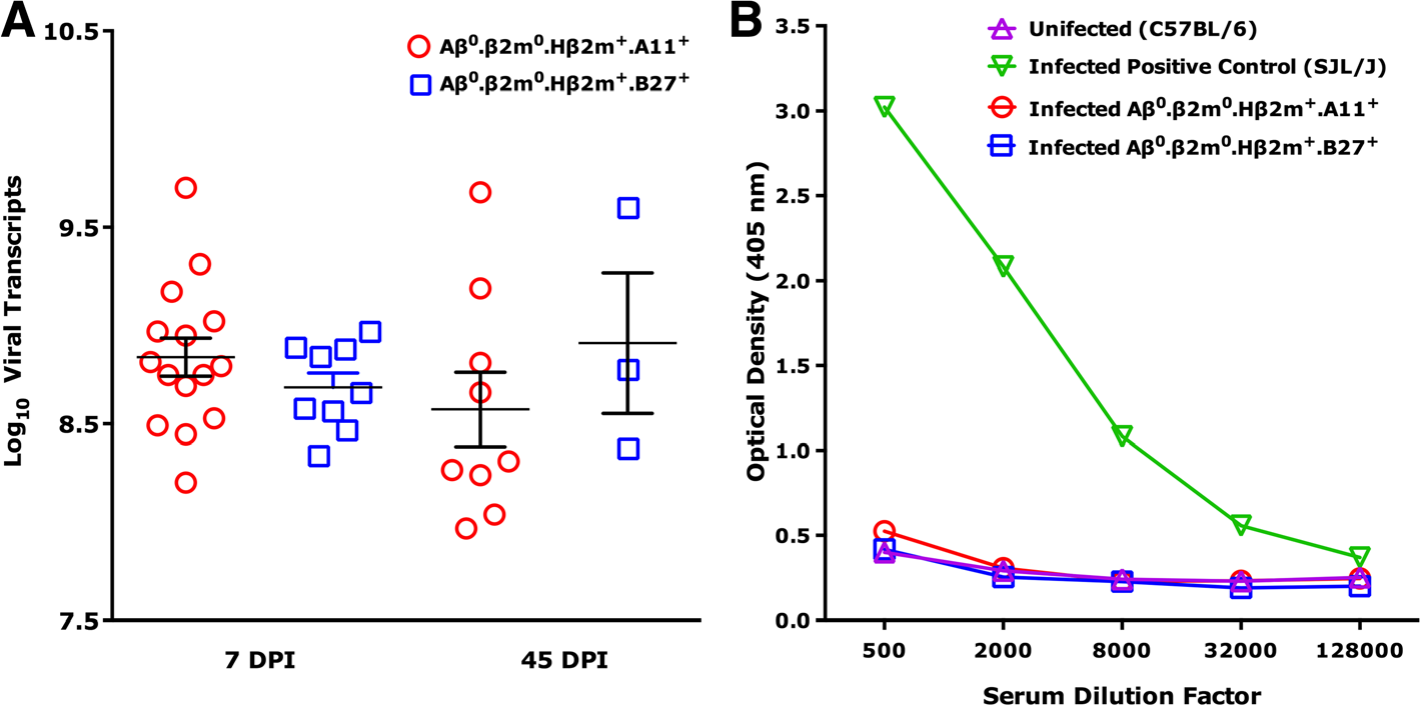
**a** Virus RNA expression. Levels of virus RNA expression in mice at 7, 21, and 45 dpi analyzed in the brain. Levels of viral capsid VP2 RNA message were quantified by light cycler PCR. There were no significant differences between the two strains in the levels of virus RNA expression at 7, 21, and 45 dpi. The levels of expression of GAPDH RNA were consistent among the groups (brains log_10_ 7.40 ± 0.03). **b** Virus-specific humoral immune responses. ELISA for serum IgG antibodies at 45 days after infection directed against purified TMEV antigens in Aβ^0^.β2m^0^.Hβ2m^+^.A11^+^and Aβ^0^.β2m^0^.Hβ2m^+^.B27^+^ mice. Uninfected C57BL/6 mice were used as a negative control. As a positive control, immunocompetent SJL/J mice were used. Five animals were tested in each experimental group

Previous reports indicate that following TMEV infection, viral RNA persists during chronic stages of the disease, even though it is often difficult to detect infectious virus by plaque assay [45]. We developed a sensitive and quantitative RT-PCR assay to measure the copy number of TMEV-specific RNA in the brain and spinal cord of infected mice (Fig. 6a). This assay has shown positive correlation with virus plaque assay [50]. We evaluated the level of virus RNA expression in the brain independently at 7 dpi (Aβ^0^.β2m^0^.Hβ2m^+^.A11^+^, log_10_ 8.84 ± 0.09; Aβ^0^.β2m^0^.Hβ2m^+^.B27^+^, log_10_ 8.84 ± 0.09) and 45 dpi (Aβ^0^.β2m^0^.Hβ2m^+^.A11^+^, log_10_ 8.84 ± 0.09; Aβ^0^.β2m^0^.Hβ2m^+^.B27^+^, log_10_ 8.91 ± 0.35). We focused our experiments primarily to test whether the persistence of severe brain pathology in Aβ.β2m^0^.Hβ2m^+^.A11^+^ mice was a consequence of more virus RNA propagation. All experiments were controlled for expression of GAPDH RNA, which was highly consistent among different strains. The level of GAPDH-RNA in the brain was log_10_ 7.40 ± 0.03.

As expected, all mice strains expressed high levels of virus RNA in the brain during acute infection. Given the absence of a protective immune response, more virus RNA copies (log_10_ 12.28 ± 0.06) were detected in Aβ^0^.β2m^0^ mice compared to the other strains. The difference was statistically significant when virus RNA copy numbers were compared among all strains for the brain (*P* < 0.001, one-way ANOVA). We used the Tukey test for multiple-pair comparisons of virus copy numbers in Aβ^0^.β2m^0^ mice relative to virus copy numbers in the other strains. The viral copy number was not statistically significant when comparing Aβ^0^.β2m^0^.Hβ2m^+^.A11^+^ mice to Aβ^0^.β2m^0^.Hβ2m^+^.B27^+^ mice at neither 7 nor 45 dpi (Fig. 6a). This indicates that, in the absence of Aβ (class II immune response), human class I worked efficiently to limit virus propagation and prevent death and subsequent injury. Most notable was the comparison of virus-RNA at the 45 dpi, when Aβ^0^.β2m^0^.Hβ2m^+^.A11^+^ mice showed severe brain pathology but Aβ^0^.β2m^0^.Hβ2m^+^.B27^+^ mice had repaired the injury. There was no difference in the amount of virus RNA when comparing Aβ^0^.β2m^0^.Hβ2m^+^.A11^+^ mice to Aβ^0^.β2m^0^.Hβ2m^+^.B27^+^ mice at 7 or 45 dpi. Therefore, clearance of virus RNA did not explain the effect of the human class I MHC on differential brain repair.

### Humoral immune responses are not altered in Aβ^0^.β2m^0^.Hβ2m^+^.A11^+^ and Aβ^0^.β2m^0^.Hβ2m^+^.B27^+^ transgenic mice following TMEV infection

Antibody production is an efficient way to prevent and control viral infection by the immune system [51]. Therefore, we asked whether phenotypic differences between Aβ^0^.β2m^0^.Hβ2m^+^.A11^+^ mice and Aβ^0^.β2m^0^.Hβ2m^+^.B27^+^ mice were at least partially based on differences in the humoral response to the virus. To test this possibility, we assessed antibody responses in the serum by ELISA directed against purified virus antigens. Serum IgG responses were measured at 21 (not shown) and 45 dpi (Fig. 6b). Neither strain of mice developed virus-specific IgG responses at 21 and 45 dpi. Results from TMEV-infected strains were identical to IgG levels observed in non-infected littermate controls, which showed no antivirus antibody responses. In contrast, antibody responses from immunocompetent mice (SJL/J) showed strong positive reactivity by ELISA to virus antigen. The lack of an Aβ gene in these transgenic mice explained the absence of virus-specific antibody responses because the class II MHC immune response is necessary for the B cell-mediated antibody generation. Therefore, a protective antibody response does not account for phenotypic differences seen between Aβ^0^.β2m^0^.Hβ2m^+^.A11^+^ and Aβ^0^.β2m^0^.Hβ2m^+^.B27^+^ mice with respect to their regenerative ability in hippocampus and striatum.

## Discussion

To our best knowledge, this is the first study to demonstrate human class I MHC gene-mediated survival and modulation of viral infectivity in a transgenic mouse model devoid of a class II immune response. Transgenic expression of either A11 or B27 prevented the phenotypic consequence of persistent viral infection; in this case, chronic demyelination. Of interest, the extent of pathology observed in Aβ^0^.β2m^0^.Hβ2m^+^.A11^+^ or Aβ^0^.β2m^0^.Hβ2m^+^.B27^+^ mice was less than that observed in Aβ^0^ mice with endogenous mouse class I H-2^*b*^ alleles. This unexpected finding suggests that the human A11 or B27 gene in this scenario functions even more efficiently than the endogenous murine class I H-2^*b*^ alleles in limiting early pathologic damage. This occurred despite an otherwise murine-immune repertoire with mouse T cells and mouse T cell receptors. These findings support the hypothesis that human class I molecules are critical for controlling the extent of pathologic damage following intracerebral virus infection.

Previous studies demonstrate that class I-deficient (β2m^0^) [52–54] or class II-deficient (Aβ^0^) [47, 55] mice survive encephalitic phase, but, over time, develop spinal cord demyelination. These results were confirmed in the present study. The results from the β2m^0^ or Aβ^0^ mice indicate that neither independent CD4^+^ nor CD8^+^ T cells are required for early limitation of virus replication in the brain, since these mice survive the acute infection. However, both strains of mice fail to clear the virus completely, and therefore, persistent infection remains in the spinal cord. In addition, the results from the β2m^0^ or Aβ^0^ mice indicate that either class I-restricted or class II-restricted T cells are required independently for subsequent demyelination. The use of CD4 and CD8 knockout mice confirms this finding [56]. This is in contrast to the infection of double deficient Aβ^0^.β2m^0^ mice, which fail to clear the acute virus encephalitis and die possibly before demyelination occurs in the spinal cord [48]. In the present experiment, the disease in Aβ^0^.β2m^0^.Hβ2m^+^.A11^+^ and Aβ^0^.β2m^0^.Hβ2m^+^.B27^+^ mice was similar to the established model. These mice had substitution of mouse class I alleles with human A11 or B27 genes, and thus, they survived acute encephalitis similarly to Aβ^0^ mice. In addition, these transgenic mice were similar to Aβ^0^ mice [57] regarding the virus-specific antibody responses, because none of the A11^+^, B27^+^ or the Aβ^0^ mice developed antibody responses.

We considered two hypotheses to explain the differences in brain pathology and repair as a result of human class I expression following chronic infection. One possibility was that a particular class I immune response influenced more injury to brain neurons. In this scenario, the presence of A11+ transgene would actively contribute to injury, whereas the reparative response in Aβ^0^.β2m^0^.Hβ2m^+^.B27^+^ transgenic mice was passive, and thus occurred independently of the MHC haplotype. The other possibility was that the reparative response in the Aβ^0^.β2m^0^.Hβ2m^+^.B27^+^ transgenic mice was active, such that a protective response was necessary for neuronal repair dependent of human class I MHC. To address these two possibilities, we tested the reparative response in Aβ^0^.β2m^0^.Hβ2m^+^ mice, which expressed only Hβ2m but no specific class I MHC allele. Of importance, these mice showed CNS repair following virus infection. The brain pathologic score for Aβ^0^.β2m^0^.Hβ2m^+^ mice at 45 days after infection was 0.429 ± 0.104 (*N* = 77). There was a statistical trend (*P* = 0.070, rank sum test) for less brain pathology in Aβ^0^.β2m^0^.Hβ2m^+^ mice as compared to Aβ^0^.β2m^0^.Hβ2m^+^.A11^+^ mice (0.964 ± 0.137, *N* = 112). There was no statistical difference (*P* = 0.641, rank sum test) between the brain pathology in Aβ^0^.β2m^0^.Hβ2m^+^ mice as compared to Aβ^0^.β2m^0^.Hβ2m^+^.B27^+^ mice (0.403 ± 0.198, *N* = 77). This supports the hypothesis that brain repair was an active process since mice with only Hβ2m^+^ showed some repair. The second hypothesis was that the class I MHC or possibly even Hβ2m alone may have provided a signal that promoted repair. However, since the differences for brain pathology comparing Aβ^0^.β2m^0^.Hβ2m^+^ mice did not reach statistical significance, we consider this less likely.

However, the most striking aspect of these studies was the difference in how human MHC class I genes influenced repair or persistent brain damage after the initial injury. Infection of Aβ^0^.β2m^0^.Hβ2m^+^.A11^+^ and Aβ^0^.β2m^0^.Hβ2m^+^.B27^+^ mice resulted in a similar extent of damage in the hippocampus, striatum, and cortex at 7 dpi. In particular, there was striking loss of pyramidal cells of the hippocampus with evident necrosis and loss of architecture. However, at 45 dpi, Aβ^0^.β2m^0^.Hβ2m^+^.B27^+^ mice showed remarkable repair of these structures, whereas Aβ^0^.β2m^0^.Hβ2m^+^.A11^+^ mice featured persistent injury. A very likely explanation for these findings is the control of virus antigen expression or virus RNA in the Aβ^0^.β2m^0^.Hβ2m^+^.B27^+^ mice but persistent virus infection in Aβ^0^.β2m^0^.Hβ2m^+^.A11^+^ mice. Surprisingly, we found that both strains of mice control virus infection such that minimal virus antigen-positive cells were quantified in the brain at 45 dpi. In addition, virus RNA transcripts were not different in the brain in these two strains. There was also a lack of demyelination in the spinal cord during chronic infection. Thus, the differential effect on repair cannot be explained by failure of MHC class I presentation in the A11^+^ strain resulting in virus persistent injury. In contrast, the data support the hypothesis that repair occurs independently of viral persistence and is an inherent property of the interaction between the nervous system and the class I MHC. One important limitation is that only one transgenic line from each of the Aβ^0^.β2m^0^.Hβ2m^+^.A11^+^ and Aβ^0^.β2m^0^.Hβ2m^+^.B27^+^ mice were used to derive our data. We cannot exclude the possibility that there is another gene, closely linked to the MHC genes, used to make these mice as the reason for the brain repair observed in the Aβ^0^.β2m^0^.Hβ2m^+^.B27^+^ mice. We await confirmation of these results by other investigators.

What are the potential mechanisms by which the MHC I class arm of the immune response contributes to brain repair? Previous conventional wisdom taught that immune factors are detrimental to the CNS. However, recent data indicate a critical role in CNS repair for certain aspects of the innate [58] and adaptive immune responses [59]. Increasing evidence implies that T cells are necessary for CNS repair. Studies from our laboratory using immune RAG-1-deficient mice point to failure of remyelination following toxin-induced demyelination [60]. Mice lacking or depleted of CD4^+^ or CD8^+^ T cells also exhibit reduced remyelination. Others have demonstrated that immune-activated T cells and macrophages are beneficial in spinal cord injury [61]. Immune cells actively secrete trophic factors to promote CNS repair [62]. These factors, including interferon gamma [63], interleukin 6 [41, 64], and TNF [65, 66], are secreted by T cells and macrophages and promote neuronal differentiation and neurite outgrowth. Many of these factors promote neuronal survival, neuronal stem cell proliferation, neurite elongation, or prevent cell death in vitro. Differential MHC class I expression in our transgenic mice may have altered neural stem cell or progenitor recruitment. For example, there is evidence that neural stem cells express co-stimulatory molecules (i.e., CD80 and CD87) differentially regulated by the inflammatory response [67]. Furthermore, implantation of immune-activated dendritic cells in the injured adult spinal cord may activate endogenous neural stem cells leading to de novo neurogenesis [68, 69]. Understanding how differential expression of human class I MHC mediates repair is beyond the scope of this paper and will require extensive investigation.

## Conclusions

Based on the results from this study, we propose the following model to explain the effect of differential class I MHC alleles on CNS injury or repair. In the absence of an immune response (i.e., Aβ^0^.β2m^0^ mice), animals die of overwhelming encephalitis as a result of widespread infection. The presence of either class II (i.e., Aβ^0^ mice) or class I (i.e., β2m^0^ mice) immune response is sufficient to overcome the fatal encephalitis. These mice clear the virus from neurons in the brain but develop virus persistence in glial cells and macrophages leading to chronic spinal cord demyelination. In the presence of both a competent class I and class II response (i.e., C57Bl/6 – H2^*b*^ mice), virus is cleared without subsequent demyelination or virus persistence. Differential class I alleles appear to play a critical role in determining whether the final outcome of injury is persistent neuronal dropout (i.e., Aβ^0^.β2m^0^.Hβ2m^+^.A11^+^ mice) or neural repair (i.e., Aβ^0^.β2m^0^.Hβ2m^+^.B27^+^ mice).

## Additional file

Additional file 1: Figure S1. Spinal cord lesions in mice infected for 45 days with DA strain of TMEV. The class I antigen expression was up-regulated in areas of injury. Panel A. Immunoperoxidase A11 staining in Aβ^0^.β2m^0^.Hβ2m^+^.A11^+^ mice. Panel B. B27 staining in Aβ^0^.β2m^0^.Hβ2m^+^.A11^+^ mice. Panel C. B27 staining in Aβ^0^.β2m^0^.Hβ2m^+^.B27^+^ mice. Panel D. A11 staining in Aβ^0^.β2m^0^.Hβ2m^+^.B27^+^ mice. Images were collected at ×60. The weak staining is either the result of the quality of antibodies that do not work well with immunocytochemistry or the fact that the CNS normally has low level expression of MHC. Scale bar = 20 μm. (TIF 1561 kb)

## Abbreviations

ANOVA: Analysis of variance
Aβ^0^ mice: Mice lacking functional MHC class II molecules
DNA: Deoxyribonucleic acid
DPI: Days post-infection
ELISA: Enzyme-linked immunosorbent assay
FACS: Fluorescence-activated cell sorting
GAPDH: Glyceraldehyde 3-phosphate dehydrogenase
h.p.f.: High-power field
HLA: Human leukocyte antigen
Hβ2m: Human beta-2 microglobulin
MHC: Major histocompatibility complex
PCR: Polymerase chain reaction
RNA: Ribonucleic acid
RT-PCR: Reverse transcription polymerase chain reaction
TMEV: Theiler’s murine encephalomyelitis virus
β2m: MHC class I molecule, i.e., beta-2 microglobulin
