# Supplementary figures and images for: Human class I major histocompatibility complex alleles determine central nervous system injury versus repair
Source: J Neuroinflammation. 2016 Nov 17;13:293. doi: 10.1186/s12974-016-0759-4 (PMC5112886; doi:10.1186/s12974-016-0759-4)

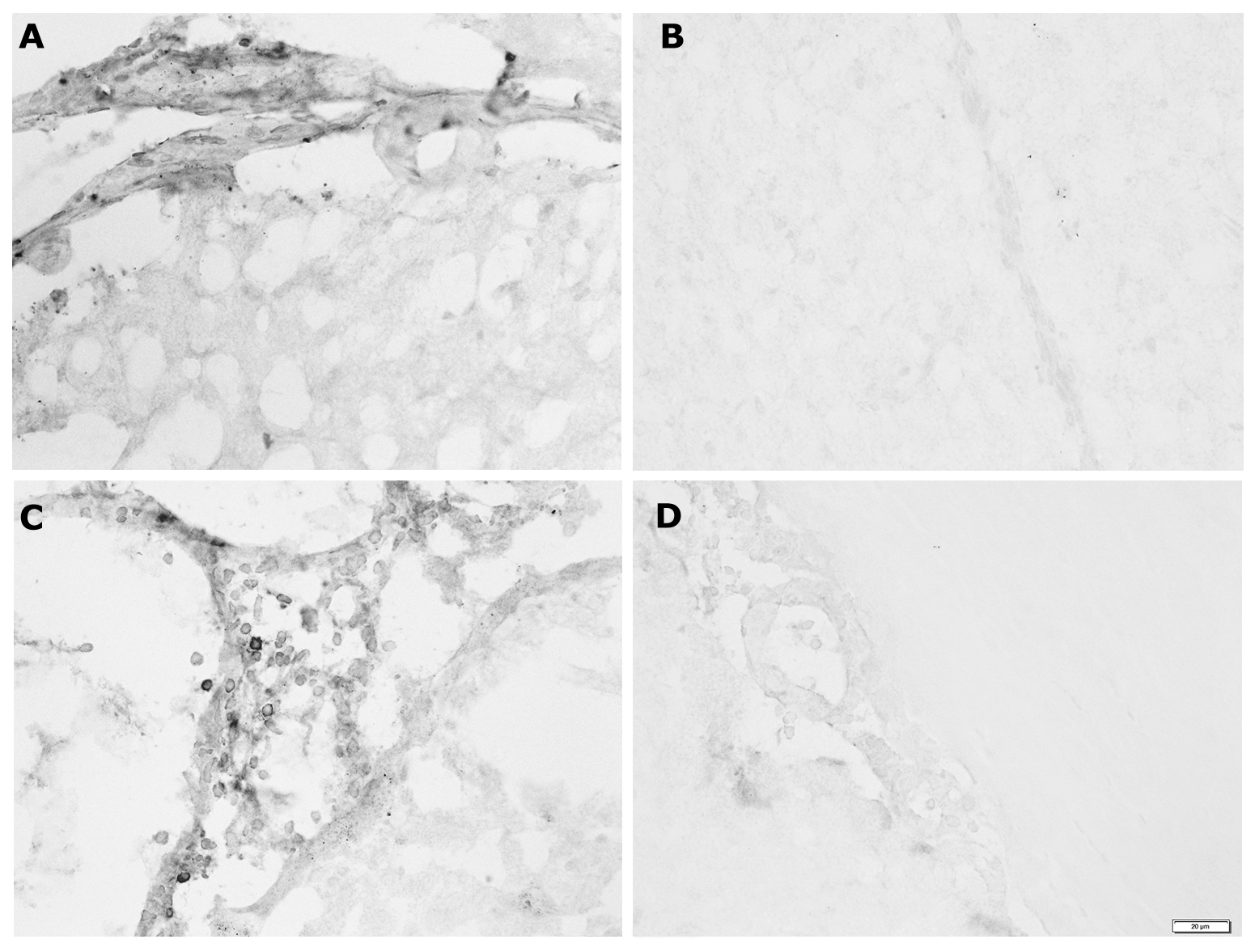

Supplement: Additional file 1: Figure S1. — Spinal cord lesions in mice infected for 45 days with DA strain of TMEV. The class I antigen expression was up-regulated in areas of injury. Panel A. Immunoperoxidase A11 staining in Aβ0.β2m0.Hβ2m+.A11+ mice. Panel B. B27 staining in Aβ0.β2m0.Hβ2m+.A11+ mice. Panel C. B27 staining in Aβ0.β2m0.Hβ2m+.B27+ mice. Panel D. A11 staining in Aβ0.β2m0.Hβ2m+.B27+ mice. Images were collected at ×60. The weak staining is either the result of the quality of antibodies that do not work well with immunocytochemistry or the fact that the CNS normally has low level expression of MHC. Scale bar = 20 μm. (TIF 1561 kb) [file 12974_2016_759_MOESM1_ESM.tif]
